# *De Novo* Purine Biosynthesis Is Required for Intracellular Growth of Staphylococcus aureus and for the Hypervirulence Phenotype of a *purR* Mutant

**DOI:** 10.1128/IAI.00104-20

**Published:** 2020-04-20

**Authors:** Mariya I. Goncheva, Ronald S. Flannagan, David E. Heinrichs

**Affiliations:** a Department of Microbiology and Immunology, University of Western Ontario, London, Ontario, Canada; University of California, Davis

**Keywords:** *Staphylococcus aureus*, intracellular pathogen, pathogenesis, purines

## Abstract

Staphylococcus aureus is a noted human and animal pathogen. Despite decades of research on this important bacterium, there are still many unanswered questions regarding the pathogenic mechanisms it uses to infect the mammalian host. This can be attributed to it possessing a plethora of virulence factors and complex virulence factor and metabolic regulation. PurR, the purine biosynthesis regulator, was recently also shown to regulate virulence factors in S. aureus, and mutations in *purR* result in derepression of fibronectin binding proteins (FnBPs) and extracellular toxins, required for a so-called hypervirulent phenotype. Here, we show that hypervirulent strains containing *purR* mutations can be attenuated with the addition of purine biosynthesis mutations, implicating the necessity for *de novo* purine biosynthesis in this phenotype and indicating that S. aureus in the mammalian host experiences purine limitation. Using cell culture, we showed that while *purR* mutants are not altered in epithelial cell binding, compared to that of wild-type (WT) S. aureus, *purR* mutants have enhanced invasion of these nonprofessional phagocytes, consistent with the requirement of FnBPs for invasion of these cells. This correlates with *purR* mutants having increased transcription of *fnb* genes, resulting in higher levels of surface-exposed FnBPs to promote invasion. These data provide important contributions to our understanding of how the pathogenesis of S. aureus is affected by sensing of purine levels during infection of the mammalian host.

## Abstract (precis)

Staphylococcus aureus is a noted human and animal pathogen. Despite decades of research on this important bacterium, there are still many unanswered questions regarding the pathogenic mechanisms it uses to infect the mammalian host. This can be attributed to it possessing a plethora of virulence factors and complex virulence factor and metabolic regulation. PurR, the purine biosynthesis regulator, was recently also shown to regulate virulence factors in S. aureus, and mutations in *purR* result in derepression of fibronectin binding proteins (FnBPs) and extracellular toxins, required for a so-called hypervirulent phenotype.

## INTRODUCTION

Staphylococcus aureus is a Gram-positive bacterium that is found as a commensal in about a third of the human population (1). However, S. aureus can also be pathogenic, causing a wide array of diseases, ranging from mild skin and soft tissue infections to life-threatening infections such as endocarditis, pneumonia, and bacteremia (2). Data demonstrating that morbidity and mortality due to invasive S. aureus infection in the United States cause more deaths than HIV (3) lend further support to the burden that S. aureus infections place on society.

Purines are essential to life. All organisms, except for some parasitic worms, can synthesize purines *de novo*. In S. aureus, *de novo* purine biosynthesis is accomplished by the activity of 11 enzymes that convert phosphoribosyl pyrophosphate (PRPP) to IMP (see Fig. S1A in the supplemental material). IMP can then be converted to ATP or GTP by the PurA and PurB or the GuaA and GuaB proteins, respectively. Previous reports have shown that *de novo* purine biosynthesis is required for full virulence of Francisella tularensis (4), Brucella abortus (5), Escherichia coli (6), and many other pathogens. In S. aureus strain Newman, *purA* and *purH* mutants are attenuated *in vivo* (7). Furthermore, S. aureus with mutations in *guaA* or *guaB* cannot grow in serum and fail to establish infection in a murine model (8). A *purF* mutant of USA300 was shown to have a modest defect in a rabbit endocarditis model, but the *purF* mutation did render the bacterium highly susceptible to vancomycin treatment (9).

Recently, it was demonstrated that inactivation of the transcriptional repressor of purine biosynthesis, PurR, results in hypervirulent S. aureus in a mouse bacteremia model (10, 11). In *purR*-deficient S. aureus, transcription of purine biosynthesis genes and known virulence factor genes, including those encoding fibronectin binding proteins (FnBPs), is increased (10, 11). This *purR* mutant-dependent hypervirulent state was found to be mediated by aberrant upregulation of FnBPs, whose expression is normally repressed by PurR. Since several known virulence factors, including exotoxins (11), are controlled by PurR, it is unclear whether FnBP expression alone is sufficient for hypervirulence of *purR* S. aureus or whether the concurrent substantial increase in *pur* gene transcription is also required. Moreover, the specific events that occur *in vivo* that lead to increased virulence are unknown.

As FnBPs are required for the invasion of nonphagocytic cells by S. aureus (12–14), we sought to determine if *purR* mutants demonstrate increased invasion, which could in part account for their increased pathogenesis. Furthermore, we hypothesized that the increase in *de novo* purine biosynthesis may confer a growth advantage during intracellular replication in macrophages, allowing faster escape of *purR* mutant S. aureus from Kupffer cells and quicker dissemination to other organs. Here, we demonstrate that *purR*-deficient S. aureus has an increased capacity to invade epithelial cells and concurrently requires *de novo* purine biosynthesis for intracellular replication in the absence of exogenous purines. Moreover, a systemic murine infection model mirrors these findings and demonstrates that the ability to synthesize purines *de novo* is essential for the pathogenesis of *purR*-deficient S. aureus, regardless of increased FnBP expression.

## RESULTS

### *De novo* purine biosynthesis is required for S. aureus replication and pathogenesis *in vivo*.

**FIG 1:**
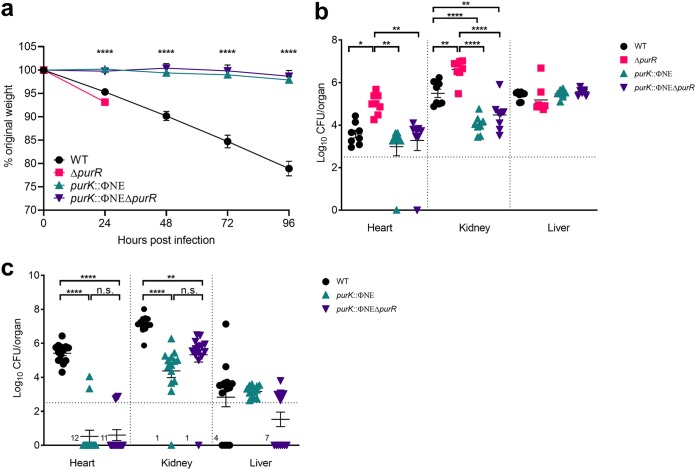
*De novo* purine biosynthesis is required for S. aureus pathogenesis *in vivo.* Groups of 6 to 8 8-week-old female BALB/c mice were infected with 1 × 10^7^ CFU of S. aureus via tail vein injection. (a) Animal weight was recorded daily and is shown as percentage of weight loss from initial weight. Data are means ± SEMs from 2 experiments with a total of 14 animals. (b) At 24 hpi animals were sacrificed, organs harvested, and CFU per organ determined. Data are means ± SEMs for 8 animals per group. The dotted line represents the limit of accurate detection. (c) At 96 hpi animals were sacrificed, organs harvested, and CFU per organ determined. Data are means ± SEMs from 2 experiments with a total of 14 animals. The dotted line represents the limit of accurate detection. Animals infected with the Δ*purR* mutant met early euthanasia criteria at 24 hpi. *, *P* value < 0.05; **, *P* value < 0.01; ****, *P* value < 0.0001, based on one-way analysis of variance (ANOVA) with Bonferroni posttest.

Previously, we showed that FnBPs are essential for the hypervirulence of a *purR* mutant (10). However, it was not known whether FnBP expression is sufficient for this phenotype or whether the concurrent increase in *pur* gene expression contributes to rapid lethality in mice. In an attempt to address this at the outset of this study, we assessed the virulence of an S. aureus USA300 *purK*::ΦNΣ mutant (15) (the mutation results in a block in the purine biosynthesis pathway [Fig. S1]), along with a *purK*::ΦNΣ Δ*purR* double mutant (see below), in relation to those of the wild type (WT) and a Δ*purR* mutant. To do this, we infected mice intravenously (i.v.) with each of the four strains using a well-established model of murine bacteremia. While WT-infected animals steadily lost weight over the course of the 4 days of infection, animals infected with the *purR* mutant required sacrifice at 24 h postinfection (hpi), as previously demonstrated (10) (Fig. 1a), and this correlated with significant increases in bacterial burden, versus those of the WT, in the heart and kidneys at 24 hpi (Fig. 1b). In contrast, animals infected with the *purK* mutant did not lose weight (Fig. 1a) or show outward signs of disease, even by 96 hpi, and had significantly lower bacterial burdens in the heart and kidneys (Fig. 1c). Most importantly, we found that including the *purK*::ΦNΣ mutation in the Δ*purR* strain converted what was a hypervirulent *purR* mutant strain into an attenuated strain (Fig. 1). In fact, not only was removal of *de novo* purine biosynthesis sufficient to eliminate the hypervirulence of a *purR* mutant, but also it reduced bacterial virulence, as evidenced by weight loss and bacterial burdens, to levels lower than those of the WT. Together, these data demonstrate that *de novo* purine biosynthesis is required for the pathogenesis of S. aureus, as well as for the hypervirulence associated with *purR* inactivation during systemic disease.

### Lack of *de novo* purine biosynthesis is without effect on serum- and FnBP-dependent hyperclumping of S. aureus.

**FIG 2:**
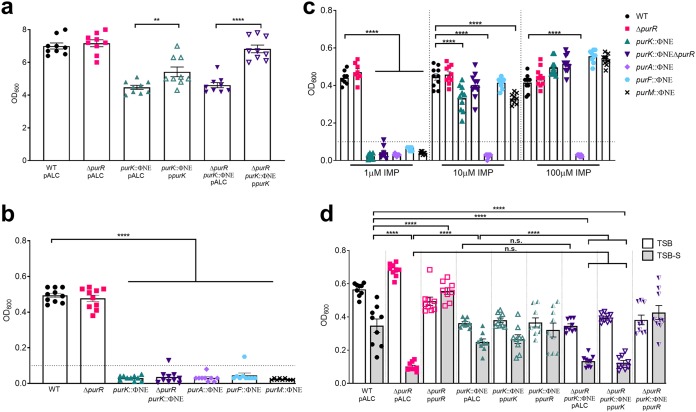
Lack of *de novo* purine biosynthesis limits growth of S. aureus but not FnBP-dependent serum clumping of Δ*purR* S. aureus. (a) S. aureus strains were grown in TSB with 200 ng/ml of tetracycline O/N at 37°C. Endpoint growth was determined by measuring OD_600_. Data are means ± SEMs from 3 independent experiments, with 3 biological replicates per experiment. (b) Strains were grown in TSB O/N, diluted to an OD_600_ of 0.01, and grown in DMEM for 24 h. Data are means ± SEMs from 3 independent experiments, with 3 biological replicates per experiment. The horizontal dotted line represents the limit of accurate detection. (c) Strains were grown in TSB O/N, diluted to an OD_600_ of 0.01, and grown in DMEM supplemented with various concentrations of IMP for 24 h. Data are means ± SEMs from 3 independent experiments, with 3 biological replicates per experiment. The horizontal dotted line represents the limit of accurate detection. (d) Strains were grown in TSB or TSB with 10% (vol/vol) horse serum (TSB-S) for 3.5 h at 37°C. Optical density of the center of the tube was measured after static incubation for 5 min. Data are means ± SEMs from 3 independent experiments, with 3 biological samples per experiment. *, *P* value < 0.05; **, *P* value < 0.01; ****, *P* value < 0.0001, based on one-way ANOVA with Bonferroni posttest.

Given that the *purK* mutation completely abrogated the hypervirulence of the *purR* mutation, we next investigated whether this may be due simply to effects on growth in the absence of purines or whether the inability to synthesize purines affected FnBP-dependent bacterial clumping in serum, which we previously correlated with hypervirulence (10). The *purK* mutant, irrespective of whether it also contained a *purR* mutation, demonstrated attenuated growth in tryptic soy broth (TSB) (Fig. 2a). Provision of *purK* in *trans* partially restored the growth defect of the single *purK* mutant, and we attribute this to the facts that *purK* is the second gene in the 11-gene operon and the transposon insertion exerts a polar effect on downstream gene transcription. Consistent with this notion, when the double mutant was complemented with the same *purK* expression plasmid, we observed full restoration of growth, ostensibly because the *purR* mutation results in significantly increased transcription of the complete *pur* operon (10).

To further investigate the requirement of *de novo* purine biosynthesis for S. aureus growth, we analyzed growth of the WT and four different purine biosynthesis gene mutants, obtained from the Nebraska transposon library (15), in a chemically defined medium lacking purines. While no differences in endpoint growth were evident between the WT and a *purR* mutant, none of the mutants that are deficient for purine biosynthesis were able to grow under identical conditions (Fig. 2b). In agreement with the idea that disruption of the purine biosynthesis pathway was the only reason for significantly diminished growth, provision of IMP restored growth of each mutant to WT levels, in a dose-dependent manner (Fig. 2c). The only exception to this was the *purA* mutant, which has a defect in the biosynthetic pathway at a step after IMP (Fig. S1) and therefore should not be supported by the presence of the metabolite. Overall, these findings demonstrate the importance of the *pur* operon for S. aureus growth. Moreover, these results demonstrate that the *purK* mutant, which is complemented by both *purK* in *trans* and by the metabolite IMP, serves as a useful mutant to study the role of purine biosynthesis in further detail, especially in the context of a *purR* mutation. Therefore, this mutant was used throughout the remainder of this study.

We next assessed whether the inability to synthesize purines inhibited the serum- and FnBP-dependent clumping phenotype of a *purR* mutant (10). As demonstrated in Fig. 2d, *purR* S. aureus displayed the characteristic hyperclumping phenotype when cultured in the presence of serum; this is characterized by a drop in culture optical density at 600 nm (OD_600_) as bacteria settle to the bottom of the culture tube within minutes. Cultures of the WT and the *purK* mutant in serum resulted in modest clumping that is characteristic of S. aureus with WT FnBP expression. In contrast, the *purR purK* S. aureus strain displayed archetypal *purR*-dependent hyperclumping, despite displaying an overall reduction in growth (Fig. 2d). This phenotype was strictly due to inactivation of *purR*, as provision of *purR* in *trans* eliminated hyperclumping, whereas provision of *purK* in *trans* did not (Fig. 2d). Taken together, these data show that in the context of a *purR* mutation, disruption of purine biosynthesis does not abrogate FnBP-dependent hyperclumping, indicating that elevated FnBP expression due to loss of *purR* is without effect.

### An S. aureus *purR* mutant demonstrates enhanced invasion of nonprofessional phagocytes.

As a way to explain the hypervirulence, we hypothesized that *purR* mutants have an increased capacity to invade nonprofessional phagocytes, since it is well established that S. aureus uses FnBPs as a means to invade these cells (12–14). We have demonstrated *purR* mutants overexpress FnBPs, at least transiently, in early stages of growth (10). To define this, we performed an invasion assay using the human lung epithelial cell line A549.

**FIG 3:**
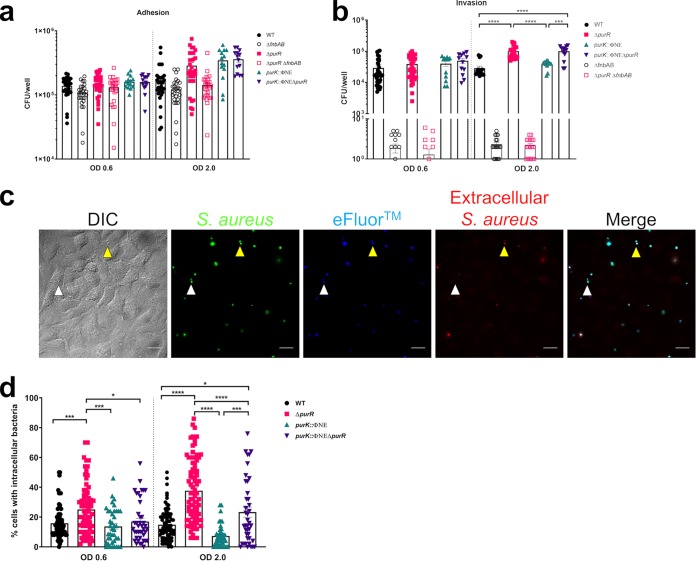
A *purR* mutant demonstrates enhanced invasion of epithelial cells. (a) Bacteria were grown to different ODs as indicated and used in gentamicin protection assays. Adhesion is shown as the total number of bacteria recovered at 30 min postinfection, prior to gentamicin treatment. Data are means ± SEMs from 5 to 8 independent experiments, with 2 biological replicates per experiment. (b) Invasion is shown as the total number of bacteria recovered at 1 h postinfection, immediately after the removal of gentamicin. Data are means ± SEMs from 5 to 8 independent experiments, with 2 biological replicates per experiment. (c) Coverslips of cells were infected with bacteria as for panel a and stained after gentamicin treatment. At onset of infection, cells were stained with eFluor 670 dye and prior to fixation were incubated with a Cy3-conjugated rabbit anti-sheep IgG to detect extracellular bacteria. Representative images of WT bacteria are shown. White arrowheads indicate intracellular bacteria; yellow arrowheads indicate extracellular bacteria. Scale bar equals 20 μm. (d) Cells in images from panel c were analyzed, and the number of epithelial cells containing bacteria was counted. Cells with extracellular bacteria were excluded. The percentage of cells with intracellular bacteria per field of view was calculated. A total of 35 to 40 fields of view of 3 or 4 independent experiments were analyzed per group. Data are means ± SEMs. *, *P* value < 0.05; ***, *P* value < 0.001; ****, *P* value < 0.0001, based on one-way ANOVA with Bonferroni posttest.

Using this system, we assessed the ability of WT or *purR*-deficient S. aureus to adhere to and invade A549 cells. Bacteria were grown to two different growth phases: an OD_600_ of 0.6, at which FnBP expression is elevated in the *purR* mutant compared to that in the WT, and an OD_600_ of 2.0, at which no transcriptional differences in *fnb* genes were previously reported (10). We observed no obvious trends in bacterial adhesion outside of a small decrease in the adhesive capacity of strains lacking FnBPs (Fig. 3a). When bacteria were grown to an OD_600_ of 0.6, mutants lacking FnBPs showed no invasion, confirming that entry into epithelial cells absolutely depends on FnBP expression. At this growth phase, we observed no significant increase in the ability of the *purR* mutant to invade epithelial cell (Fig. 3b). Interestingly, we also observed similar levels of invasion between the WT and each of the *purK* and *purR purK* mutants, further supporting our contention that the inability to synthesize purines does not affect levels of FnBP expression and, consequently, the invasive capacity of these bacteria. In contrast, when the bacteria were grown to an OD_600_ of 2.0, *purR* mutants demonstrated a significantly higher capacity to invade host cells than that of the WT (Fig. 3b, right). Furthermore, even though *purK* mutants invaded host cells similarly to the WT, the mutant lacking both *purR* and *purK* showed increased invasion over that of the WT. To confirm these data, we visualized infected A549 cells by fluorescence microscopy, using bacteria expressing green fluorescent protein (GFP) from a plasmid. We were able to determine the frequency with which each of the aforementioned strains was found inside epithelial cells (Fig. 3c and Fig. S2), using antibody-based staining to differentiate intracellular from extracellular bacteria. This analysis corroborated the results from bacterial counts, with WT and *purK* mutants demonstrating similar levels of invasion (Fig. 3d). In contrast, bacteria lacking *purR*, irrespective of the *purK* mutation, display enhanced invasion of A549 cells. Moreover, this analysis revealed that *purR* mutants are more invasive at both growth phases tested (Fig. 3d), likely because this technique is more sensitive than counting CFU. There is an apparent discordance between the growth phase-dependent transcriptional upregulation of FnBPs in *purR*-deficient S. aureus and the growth phase during which we saw increased invasion. This could be due to the effect of *purR* deficiency on other proteins that may affect FnBP expression. In S. aureus FnBPs have been shown to be posttranslationally targeted and removed from the bacterial cell surface by the action of secreted proteases, including aureolysin (16) and V8 (17). Interestingly, secreted proteases have decreased transcription in a *purR* mutant of S. aureus (10, 11), indicating that differential posttranslational regulation of surface-exposed FnBPs may occur in *purR* mutants. Together, these data demonstrate that the hyperinvasive phenotype of a *purR* mutant is due to an early transcriptional increase in FnBP expression, followed by an effect on proteins that decrease the levels of FnBPs on the bacterial cell surface.

### Purine biosynthesis mutants demonstrate decreased replication in epithelial cells.

**FIG 4:**
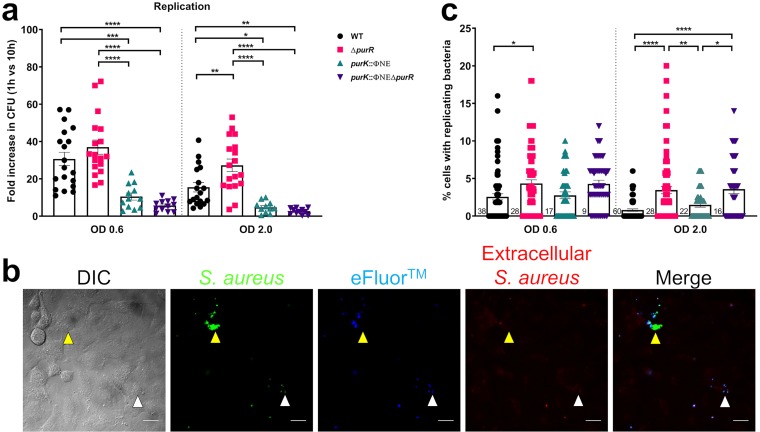
Purine biosynthesis mutants are defective for intracellular replication. A549 cells were infected as for Fig. 3 and the infection was allowed to proceed for 9 h after gentamicin removal. (a) Fold increase in bacterial numbers was calculated by dividing the number of bacteria recovered at 10 h postinfection by the number of bacteria recovered at 1 h (post-gentamicin removal). Data are means ± SEMs from 5 to 8 independent experiments, with 2 biological replicates per experiment. (b) Coverslips of cells were infected with bacteria as for Fig. 2 and were stained at 10 hpi. At onset of infection, cells were stained with eFluor 670 dye and prior to fixing were incubated with TRITC-conjugated rabbit anti-sheep IgG to detect extracellular bacteria. Representative images of WT bacteria are shown. White arrowheads indicate bacteria that have not replicated; yellow arrowheads indicate bacteria that have replicated intracellularly (extracellular bacteria are stained red). Scale bar equals 20 μm. (c) Cells in images from panel b were analyzed, and the number of epithelial cells containing bacteria was counted. Cells with eFluor 670-negative bacteria (white arrowheads in panel b), indicating intracellular replication, were also counted, and the percentage of cells with intracellular replicating bacteria per field of view was calculated. A total of 35 to 40 fields of view of 3 or 4 independent experiments were analyzed per group. Data are means ± SEMs. *, *P* value < 0.05; **, *P* value < 0.01; ***, *P* value < 0.001; ****, *P* value < 0.0001, based on one-way ANOVA with Bonferroni posttest.

The invasive capacity of S. aureus is strictly dependent on FnBPs, but the ability to replicate intracellularly is a multifactorial process. We speculated that the increased pool of purines in a *purR* mutant may provide an advantage in the restricted intracellular environment. Therefore, we sought to determine the ability of S. aureus to replicate in epithelial cells. Examination of bacterial burden at different times postinfection demonstrated that replication began by approximately 8 hpi; thus, we routinely measured intracellular replication at 10 hpi for the most reliable data (Fig. S3). Replication of the *purR* mutant showed trends similar to those observed for invasion, with *purR*-deficient bacteria showing increased levels of replication when the inoculum was grown to an OD_600_ of 2.0 (Fig. 4a). To test the dependence of intracellular replication on *de novo* purine biosynthesis, we also examined the replication of the *purK* and *purR purK* mutants. We observed a marked defect in intracellular replication for both the *purK* and *purR purK* mutants (Fig. 4a), demonstrating a strict reliance on purine biosynthesis for this intracellular replication.

We sought to examine this in further detail using fluorescence microscopy. Our lab has established a fluorescence-based proliferation assay (18) in which bacteria are surface labeled with eFluor 670 at the outset of infection and lose the dye as they undergo replication. We used this system to examine intracellular replication at a single-cell level, where bacteria that have proliferated will be GFP positive and eFluor 670 negative (Fig. 4b and Fig. S4). Analysis of images acquired at 10 hpi demonstrated that more replication occurred in *purR*-infected cells than in WT-infected cells (Fig. 4c). Furthermore, despite the low-level increase in CFU, the *purK* and *purR purK* mutants were still capable of some intracellular replication (Fig. 4c). In fact, the numbers of cells containing replicating bacteria were not very different (Fig. 4c), suggesting that replication occurs for the *purK* mutant bacteria but to levels significantly lower than those seen with the WT. Overall, these data demonstrate that mutations in the *pur* pathway hinder but do not prevent the ability of S. aureus to replicate intracellularly and that a *purR* mutant shows improved intracellular replication compared to that of the WT.

### The effect of a *purR* mutation on intracellular replication is due to increased invasion.

**FIG 5:**
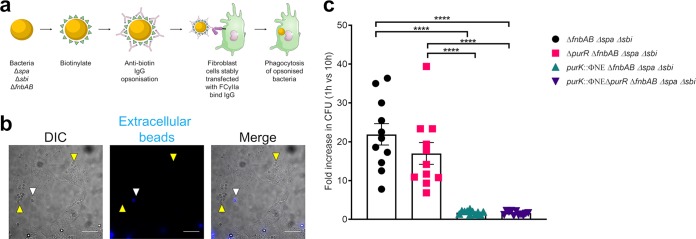
The intracellular replication of a *purR* mutant is equivalent to that of the WT when differences in cell invasion capacity are removed. (a) Schematic representation of the experiments performed with COSIIA cells. (b) Beads (0.3 μm) were opsonized with human IgG and added to COSIIA cells for 30 min. Extracellular beads were then stained with Cy5-conjugated anti-human IgG, and cells were fixed and imaged on a wide-field microscope. Representative images are shown. Scale bar equals 20 μm. (c) Indicated strains were treated according to the schematic shown in panel a and added to confluent COSIIA cells for 30 min, followed by treatment with gentamicin for 30 min. At 9 h post-gentamicin treatment, cells were lysed and plated and CFU were counted. Data are fold increase in CFU at 10 h compared to the value 1 h (immediately after gentamicin removal). Data are means ± SEMs from 5 experiments, with 2 or 3 biological replicates per experiment. ****, *P* value < 0.0001, based on one-way ANOVA with Bonferroni posttest.

In addition to increased invasion of *purR* mutants, we also saw increased intracellular replication in epithelial cells. To determine whether *purR* bacteria truly proliferate more efficiently within host cells or whether this was simply due to increased host cell invasion, we devised an experiment to circumvent FnBP-dependent bacterial uptake. To do this, we employed COS7 fibroblasts stably expressing human FcγIIa receptor (COSIIA cells), which confers on these cells the ability to phagocytose IgG-bearing targets (19) (Fig. 5a). As this cellular model requires opsonization (Fig. 5a), the bacteria employed in this study carried deletions of the *spa* and *sbi* genes, to eliminate nonspecific IgG binding. Furthermore, to eliminate any confounding effect of FnBP expression, the bacterial strains also carried deletions of the *fnbAB* genes.

At the outset, we showed that the COSIIA cells were indeed capable of phagocytosing IgG-coated beads, verifying their ability to phagocytose in an IgG-dependent manner (Fig. 5b). In order to opsonize bacteria with IgG, we performed a biotinylation step to coat the bacteria and followed that with opsonization using an antibiotin antibody. We were able to confirm biotinylation of the bacterial cell surface through fluorescent avidin staining, and IgG opsonization was confirmed via staining with a specific fluorescent secondary antibody (data not shown). Having verified opsonization of the bacteria, we then sought to examine the ability of these strains to grow intracellularly. Examination of the replicative capacity of S. aureus in these cells demonstrated that there was no appreciable difference between the replication of WT and *purR* mutant cells (Fig. 5c). In contrast, little to no replication was observed for the *purK* or *purR purK* double mutant, in agreement with the findings for epithelial cells (Fig. 5c). Taken together, these data demonstrate that *purR* mutants do not grow at an accelerated rate within host cells but rather suggest that the replicative advantage displayed by *purR* S. aureus within epithelia is due to their enhanced ability to invade more host cells.

### *De novo* purine biosynthesis is required for S. aureus replication in macrophages.

The above-described findings demonstrate a defect of *pur* biosynthesis mutants in the ability to replicate in nonprofessional phagocytes. However, it is well established that S. aureus must replicate in Kupffer cells, the resident liver macrophages, in order to establish infection in murine systemic models (20, 21). Therefore, as we have seen a growth defect of *pur* pathway mutants *in vitro* and in epithelial cells, we were interested to study their replication in macrophages, which are the bottleneck to systemic infection in mice. Our lab has a well-established gentamicin protection assay in RAW 264.7 macrophages (22), which we utilized to interrogate the ability of *purK* and *purR purK* bacteria to grow in macrophages.

**FIG 6:**
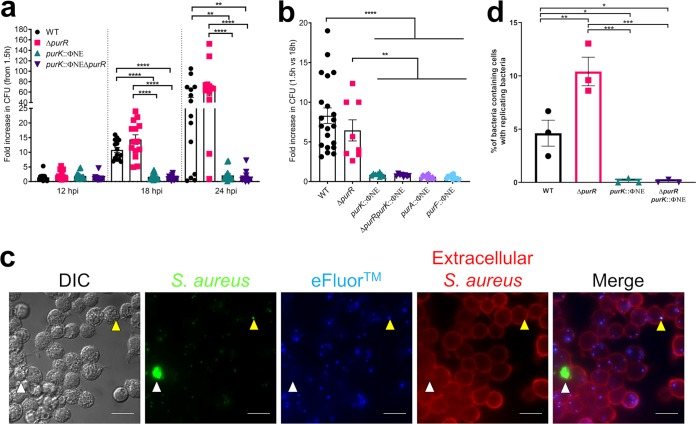
Purine biosynthesis mutants are completely attenuated in macrophages. (a) RAW 264.7 macrophages were infected with the indicated strains for 30 min, treated with gentamicin for 1 h, and maintained in RPMI 1640 plus 5% FBS. At the indicated times, cells were lysed and CFU determined. Data shown are fold increase in CFU over values recovered at 1.5 h (immediately after gentamicin treatment). Data are means ± SEMs from 5 or 6 experiments, with 2 or 3 biological replicates per experiment. (b) RAW 264.7 macrophages were infected as for panel a and lysed at 18 hpi, and CFU were determined. Data are fold increase in CFU at 18 h over values recovered at 1.5 h. Data are means ± SEMs from 4 to 6 experiments, with 2 or 3 biological replicates per experiment. (c) Bacteria were labeled with eFluor 670 and used to infect cells as for panel a. At 18 hpi the macrophage membrane was labeled with TMR wheat germ agglutinin (WGA) for 5 min and the cells were fixed. Coverslips were imaged on a wide-field microscope. Representative images are shown. Yellow arrowheads indicate bacteria that have not replicated; white arrowheads indicate bacteria that have replicated intracellularly (extracellular bacteria stain red). Scale bar equals 20 μm. (d) Cells in images from panel c were analyzed, and the cells containing bacteria and replicating bacteria were counted. The percentage of cells containing replicating bacteria was calculated by dividing the number of cells with replicating bacteria by the number of cells with bacteria. At least 10 fields of view of 3 independent experiments were analyzed. Data are means ± SEMs from. *, *P* value < 0.05; **, *P* value < 0.01; ****, *P* value < 0.0001, based on one-way ANOVA with Bonferroni posttest.

At 18 hpi intracellular replication of both WT and *purR* mutant bacteria could be seen, with no obvious differences between the two strains (Fig. 6a). In contrast, the *purK* and *purR purK* mutants showed no replication even at 24 hpi, a time by which WT and *purR* S. aureus had killed the host cells and were freely replicating in the culture medium (Fig. 6a). These data suggest that mutants unable to synthesize purines are severely restricted within professional phagocytes.

We chose the intermediate point of 18 hpi, at which intracellular replication of the WT and *purR* mutant could be reliably detected, and investigated the behavior of the purine biosynthesis mutants in more detail. To determine whether this growth impairment is specific to *purK* or if it is a general response of mutants of the *pur* pathway, we performed similar infections using *purA* and *purF* mutants. As demonstrated in Fig. 6b, intracellular growth of *purF* and *purA* mutants was also inhibited, indicating that general defects in purine biosynthesis compromise bacterial growth within macrophages. This conclusion was further supported by fluorescence imaging, which revealed that the *purK* and *purR purK* mutants failed to grow despite being phagocytosed (Fig. 6c and d and Fig. S5).

**FIG 7:**
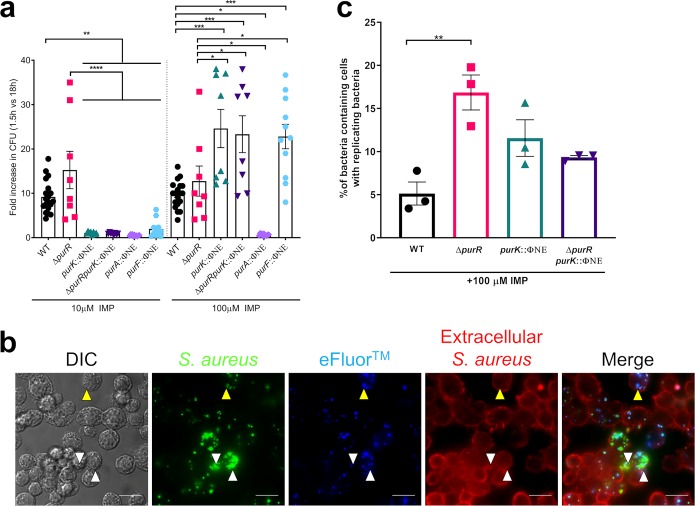
Intracellular growth defects of purine biosynthesis mutants are restored by the addition of exogenous purines. (a) RAW 264.7 macrophages were infected with the indicated strains for 30 min, treated with gentamicin for 1 h, and maintained in RPMI 1640 plus 5% FBS, with or without the indicated concentrations of IMP. At 18 hpi, cells were lysed and CFU determined. Data are fold increase in CFU over values recovered at 1.5 h. Data are means ± SEMs from 4 to 6 experiments, with 2 or 3 biological replicates per experiment. (b) Bacteria were labeled with eFluor 670 and used to infect cells as for panel a. At 18 hpi the macrophage membrane was labeled with TMR wheat germ agglutinin for 5 min, and the cells were fixed. Coverslips were imaged on a wide-field microscope. Representative images are shown. Yellow arrowheads indicate bacteria that have not replicated; white arrowheads indicate bacteria that have replicated intracellularly (extracellular bacteria stain red). Scale bar equals 20 μm. (c) Cells in images from panel b were analyzed, and the cells containing bacteria and replicating bacteria were counted. The percentage of cells containing replicating bacteria was calculated by dividing the number of cells with replicating bacteria by the number of cells with bacteria. At least 10 fields of view of 3 independent experiments were analyzed. Data are means ± SEMs. *, *P* value < 0.05; **, *P* value < 0.01; ***, *P* value < 0.001; ****, *P* value < 0.0001, based on one-way ANOVA with Bonferroni posttest.

Our findings indicate that the growth defect of purine biosynthesis mutants can be restored through the addition of exogenous purines (Fig. 2c). As we detected no replication of *pur* mutants in macrophages, we chose to assess if intracellular growth could also be rescued by supplying IMP exogenously. Indeed, the addition of 100 μM IMP to the culture medium fully restored the growth of the *purK*, *purR purK*, and *purF* mutants but not of the *purA* mutant (Fig. 7a); the *purA* mutation affects the pathway after the IMP step (Fig. S1). Analysis of fluorescence images supported these data, with replication of the *purK* and *purR purK* mutants being readily detectable in RAW 264.7 macrophages in the presence of IMP (Fig. 7 and Fig. S6). Importantly, the addition of IMP did not compromise RAW 264.7 cell viability (Fig. S7), and therefore, the observed replication was due to availability of purines, not sudden macrophage death. Indeed, exogenous IMP allowed the *purK* and *purR purK* mutants to replicate to levels higher than those of the WT (Fig. 7b and c). Together, these findings demonstrate that *de novo* purine biosynthesis is required for replication of S. aureus in macrophages.

## DISCUSSION

The ability of pathogenic bacteria to synthesize and/or acquire nutrients is integral to their survival and capacity to cause disease. Purines are essential components of life, and the importance of these macromolecules is highlighted by the fact that most free-living organisms are capable of *de novo* purine biosynthesis. Here, we demonstrate that in S. aureus, *de novo* purine biosynthesis is essential for *in vitro* and *in vivo* virulence, including hypervirulence associated with inactivation of the purine biosynthesis repressor PurR.

We report the inability of a number of *pur* mutants to grow in a chemically defined medium, which can be alleviated by the addition of exogenous purines (Fig. 2). This is consistent with previous reports for S. aureus (23), but we were interested to note that exogenous purines, while required for *pur* mutants, did not enhance the growth of WT S. aureus. Dependence on purines has also been demonstrated for Francisella tularensis (4), Brucella abortus (5), and E. coli (6), among other bacteria. S. aureus has been shown to replicate in macrophages (22, 24) and osteoclasts (25), but little is known about the nutritional requirements of S. aureus in the intracellular environment. Studies on S. aureus metabolism have identified that mutations affecting in glycolysis (*pfkA* and *pyk*) (26) and lactate dehydrogenase (27) result in reduced survival in RAW 264.7 macrophages, but no data are available on nucleotide biosynthesis. We observed decreased intracellular replication of S. aureus *pur* biosynthesis mutants in epithelial cells (Fig. 4) and virtually no replication in macrophages (Fig. 6). Interestingly, WT S. aureus has been shown to inhibit nucleotide biosynthesis of A549 cells (28), but it is presently unclear if that can influence the levels of purines in the phagosome. This is the first report demonstrating that intracellular growth of S. aureus requires purine biosynthesis, although similar findings have been reported for a number of intracellular pathogens. Indeed, *purL*, *purH*, and *purE* mutants of B. abortus have been shown to be attenuated in macrophages (5), and *purD* and *purF* mutants of the same bacterium have reported defects during replication in RAW 264.7 macrophages and HeLa cells (29). All these findings suggest the requirement for purine synthesis to grow within the intracellular environment and indicate that purines are unavailable for bacterial utilization.

Mutations in *purR* lead to hypervirulence through overexpression of the FnBPs. Nevertheless, these mutants also display upregulation of a number of other genes, including the purine biosynthesis operon (10, 11). In order to gain a better appreciation of S. aureus pathogenesis, it is critical to know whether upregulation of FnBPs alone is sufficient for this hypervirulence or whether concurrent upregulation of purine biosynthesis is also needed. We observed severe attenuation of individual purine biosynthesis mutants during murine infections, congruent with previous reports (7, 30). However, we also demonstrated similar findings for a *purR purK* mutant, which behaved like an attenuated *pur* mutant rather than the hypervirulent *purR* mutant (Fig. 1). This was further reflected in disease progression, where a *purR purK* mutant did not have improved bacterial replication later in disease.

To date, only one study has examined S. aureus with mutations in the purine biosynthetic operon and *purR*. The authors demonstrated that *purR purA* and *purR purH* mutants were more virulent than *purA* and *purH* single mutants, respectively (11). Nevertheless, in both cases, the *purR pur* mutants were still attenuated compared to WT bacteria, indicating an overall reduction in bacterial pathogenesis. Moreover, that study only examined the bacterial burden in the kidneys of infected mice at 20 hpi. We saw a similar trend in kidney samples at 24 hpi (Fig. 1b) and 96 hpi (Fig. 1c), although our data did not reach statistical significance. Therefore, our data consistently show that *de novo* purine biosynthesis is required for the establishment and progression of S. aureus bacteremia. Indeed, this fits well with our findings from macrophage infections, as replication in the resident liver macrophages (Kupffer cells) is required for the systemic spread of S. aureus from the liver (20, 21).

S. aureus is not alone in the requirement for *de novo* purine biosynthesis for full pathogenesis. There are reports of purine biosynthetic mutants of Salmonella enterica serovar Typhimurium, E. coli, and Bacillus anthracis having decreased growth in human serum (31), and S. aureus *purA* and *purB* mutants have been shown to have the same defect (23). Furthermore, animal models have demonstrated that *purF* and *purA* mutants of F. tularensis are also severely attenuated in mice (4), *purD* and *purF* mutants of B. abortus have decreased persistence (29), and a *purF* mutant of uropathogenic E. coli was attenuated in a mouse bladder colonization model (6). Overall, these findings suggest that purine availability is significantly limited during infection, and purine biosynthesis inhibitors could potentially be used in combination therapy with antibiotics to increase bacterial clearance. This is of particular importance in bacteria such as S. aureus, in which antibiotic resistance is rampant.

The ability of S. aureus to acquire nutrients is paramount to its replication and subsequent success as a pathogen. Here, we further demonstrate the essential role that *de novo* purine biosynthesis plays in the pathogenesis of S. aureus. Furthermore, we decouple the elevated expression of FnBPs and *pur* biosynthesis genes in a *purR* mutant and demonstrate their individual roles in virulence. These findings represent an important step toward the understanding of S. aureus biology during infection and the interplay between nutrient acquisition, virulence factor expression, and disease severity.

## MATERIALS AND METHODS

### Tissue culture.

Human lung epithelial A549 cells were purchased from the ATCC and maintained in Dulbecco’s modified Eagle’s medium (DMEM) with 10% (vol/vol) fetal bovine serum (FBS) at 37°C and 5% CO_2_ and passaged twice a week. RAW 264.7 macrophages were purchased from the ATCC and maintained in RPMI 1640 medium with 5% (vol/vol) FBS at 37°C and 5% CO_2_ and passaged every 2 days. COSIIA cells (19), stably transfected with FcγIIa receptor, were a gift from Sergio Grinstein and were maintained in DMEM with 10% (vol/vol) FBS and 500 μg/ml of G418 at 37°C and 5% CO_2_ and passaged twice a week.

### Bacterial growth.

Bacterial strains used in this study are listed in Table 1. E. coli was grown in Luria-Bertani (LB) broth, and S. aureus was grown in tryptic soy broth (TSB) at 37°C, shaken at 200 rpm, unless otherwise stated. Where appropriate, media were supplemented with erythromycin (3 μg/ml), chloramphenicol (12 μg/ml), lincomycin (10 μg/ml), ampicillin (100 μg/ml), or tetracycline (3 μg/ml). Solid media were supplemented with 1.5% (wt/vol) Bacto agar. For induction of complementation plasmids, bacteria were grown in TSB to an OD_600_ of 0.3 and induced with 300 ng/μl of tetracycline overnight (O/N). For growth in DMEM, bacteria were grown O/N in TSB, diluted to an OD_600_ equivalent of 0.01, and grown in 2 ml of DMEM in a 13-ml snap cap tube O/N.

**TABLE 1:** Bacterial strains used in this study

Strain	Description	Source or reference
USA300, WT	USA300 LAC, cured of resistance plasmids	Lab stock
WT pGFP	USA300 carrying superfolder GFP in plasmid pCM29 (pGFP)	This study
Δ*purR* mutant	USA300 with a deletion of *purR*	This study
Δ*fnbAB* mutant	USA300 with a deletion of *fnbAB*	10
Δ*purR* Δ*fnbAB* mutant	USA300 with deletions of *purR* and *fnbAB*	This study
Δ*purR* pGFP	USA300 with a deletion of *purR* carrying pGFP	This study
Δ*fnbAB* pGFP mutant	USA300 with a deletion of *fnbAB* carrying pGFP	This study
Δ*purR* Δ*fnbAB* pGFP mutant	USA300 with deletions of *purR* and *fnbAB* carrying pGFP	This study
*purK*::ΦΝΣ mutant	USA300 with a transposon insertion in *purK*	This study
*purK*::ΦΝΣ pGFP mutant	USA300 with a transposon insertion in *purK* carrying superfolder GFP in plasmid pCM29	This study
ΔpurR purK::ΦΝΣ mutant	USA300 with a deletion of *purR* and a transposon insertion in *purK*	This study
Δ*purR purK*::ΦΝΣ pGFP mutant	USA300 with a deletion of *purR* and a transposon insertion in *purK* carrying pGFP	This study
WT pALC	WT carrying pALC2073	10
Δ*purR* pALC mutant	USA300 with a deletion of *purR* carrying pALC2073	This study
*purK*::ΦΝΣ pALC mutant	USA300 with a transposon insertion in *purK* carrying pALC2073	This study
Δ*purR purK*::ΦΝΣ pALC	USA300 with a deletion of *purR* and a transposon insertion in *purK* carrying pALC2073	This study
Δp*urR ppurR* mutant	USA300 with a deletion of *purR* carrying a copy of *purR* in pALC2073	This study
*purK*::ΦΝΣ ppurR mutant	USA300 with a transposon insertion in *purK* carrying a copy of *purR* in pALC2073	This study
Δ*purR purK*::ΦΝΣ ppurR mutant	USA300 with a deletion of *purR* and a transposon insertion in *purK* carrying a copy of *purR* in pALC2073	This study
*purK*::ΦΝΣ ppurK mutant	USA300 with a transposon insertion in *purK* carrying a copy of *purK* in pALC2073	This study
Δ*purR purK*::ΦΝΣ ppurK mutant	USA300 with a deletion of *purR* and a transposon insertion in *purK* carrying a copy of *purK* in pALC2073	This study
*purA*::ΦΝΣ mutant	USA300 with a transposon insertion in *purA*	15
*purF*::ΦΝΣ mutant	USA300 with a transposon insertion in *purF*	15
*purM*::ΦΝΣ mutant	USA300 with a transposon insertion in *purM*	15
Δ*spa* Δ*sbi* Δ*fnbAB*	USA300 with a deletion of *spa*, *sbi*, and *fnbAB*	10
Δ*spa* Δ*sbi* Δ*fnbAB* Δ*purR* mutant	USA300 with a deletion of *spa*, *sbi*, *fnbAB*, and *purR*	This study
Δ*spa* Δ*sbi* Δ*fnbAB purK*::ΦΝΣ mutant	USA300 with a deletion of *spa*, *sbi*, and *fnbAB*, and a transposon insertion in *purK*	This study
Δ*spa* Δ*sbi* Δ*fnbAB* Δ*purR purK*::ΦΝΣ mutant	USA300 with a deletion of *spa*, *sbi*, *fnbAB*, and *purR* and a transposon insertion in *purK*	This study

### Invasion of epithelial cells.

All invasion and infection experiments were performed at a multiplicity of infection of 10. For invasion, confluent A549 cells in 12-well tissue culture plates were used. Cells were maintained in DMEM plus 10% (vol/vol) FBS until the day of infection. On the day of infection, cells were washed with PBS and maintained in serum-free (SF) DMEM for at least 1 h prior to infection. Bacterial strains of interest were grown O/N in TSB with appropriate antibiotics. Bacteria were then subcultured at an OD_600_ of 0.1 and grown in TSB with appropriate antibiotics to the desired density, as indicated throughout. Where necessary, bacteria were incubated with eFluor 670 dye (0.5 μg/ml) in PBS for 5 min, followed by the addition of TSB (18). Cells were then pelleted, washed twice with PBS, and resuspended in PBS to a density of 2 × 10^7^ CFU/ml. Fifty microliters of that suspension was added to a well of confluent A549 cells containing 700 μl of SF DMEM. Plates were pelleted at 1,000 rpm for 1 min and incubated at 37°C and 5% CO_2_ for 15 min. Cells were then washed once with PBS and fresh SF medium was added for a further 15 min at 37°C and 5% CO_2._ Cells were then treated with 150 μg/ml of gentamicin for 30 min at 37°C and 5% CO_2_, extensively washed to remove the gentamicin, and kept in SF DMEM for the desired duration of infection, as indicated throughout. At specific times postinfection, medium was removed, and cells were lysed in PBS plus 0.1% (vol/vol) Triton X-100, scraped from the well, and plated for CFU counting. For fluorescence analysis, extracellular bacteria were stained with a rabbit anti-sheep IgG conjugated to tetramethyl rhodamine isocyanate (TRITC; Jackson ImmunoResearch) (0.75 μg/ml) for 5 min, washed with PBS, and fixed with 4% (vol/vol) paraformaldehyde (PFA) for 20 min.

For infection of COSIIA cells, cells in 12-well tissue culture plates were maintained in DMEM plus 10% (vol/vol) FBS in the absence of antibiotics. On the day of the infection, cells were washed with PBS and maintained in SF DMEM for at least 1 h prior to infection. S. aureus lacking *spa*, *sbi*, and *fnbAB* were grown in TSB O/N, and 500 μl was pelleted and washed 4 times with PBS (pH 8.0). Bacteria were then resuspended in PBS (pH 8.0) with succinimidyl ester biotin and incubated at room temperature (RT) for 45 min. Cells were washed twice with PBS and incubated with mouse anti-biotin antibody (5 μg/ml; Jackson ImmunoResearch) at RT for 30 min. Cells were pelleted, washed twice with PBS, and normalized to a density of 2 × 10^7^ CFU/ml. A total of 50 μl of that suspension was added to a well of confluent COSIIA cells containing 700 μl of serum-free DMEM. Plates were pelleted at 1,000 rpm for 1 min and incubated at 37°C and 5% CO_2_ for 30 min. Cells were then treated with 150 μg/ml of gentamicin for 30 min at 37°C and 5% CO_2_, extensively washed to remove the gentamicin, and kept in SF DMEM for the desired duration of infection, as indicated throughout. At the desired times, medium was removed and cells lysed in PBS plus 0.1% (vol/vol) Triton X-100, scraped from the well, and plated for CFU determination.

### IgG bead opsonization.

Silica beads (3.14 μm; Bangs Laboratories) were opsonized with human IgG (0.8 mg/ml) for 1 h. IgG‐opsonized beads were added to individual wells containing COSIIA cells and then centrifuged at 277 × *g* for 1 min to synchronize binding of targets to the cells. After phagocytosis for 30 min, cells were washed vigorously to remove unbound silica beads. Beads remaining extracellular were detected by staining for 3 min with anti‐human fluorophore‐conjugated secondary antibodies (0.75 μg/ml) prior to fixation with 4% (vol/vol) PFA.

### Macrophage infections.

For infection of RAW 264.7 macrophages, the protocol as established by Flannagan et al. (22) was used. Where necessary, cells were supplemented with IMP after the removal of gentamicin. For propidium iodide (PI) stains, cells were treated with 1 μg/ml of PI in RPMI 1640 for 5 min prior to live-cell imaging (22). Where necessary, bacterial cells were incubated with eFluor 670 dye (0.5 μg/ml) in PBS for 5 min, followed by the addition of TSB (18). For fluorescence analysis, cells were stained with tetramethylrhodamine (TMR)-conjugated wheat germ agglutinin (WGA) (1 μg/ml) for 5 min, washed with PBS, and fixed with 4% (vol/vol) PFA for 20 min.

### Fluorescence microscopy.

Wide-field fluorescence and differential inference contrast (DIC) microscopy was performed on a Leica DMI6000 B inverted microscope equipped with 40× (numerical aperture [NA], 1.3), 63× (NA, 1.4) and 100× (NA, 1.4) oil immersion PL‐Apo objectives, a Leica 100-W Hg high-pressure light source, and Hammamatsu Orca Flash 4.0 and Photometrics Evolve 512 Delta electron-multiplying charge-coupled-device (EMCCD) cameras. All images were analyzed and contrast enhanced using ImageJ (National Institutes of Health, Bethesda, MD).

### Clumping assays.

Clumping assays were performed as previously described (10). Briefly, O/N cultures grown in TSB were diluted to an OD_600_ equivalent of 0.01 and grown in TSB or TSB with 10% (vol/vol) heat-inactivated horse serum for 3.5 h at 37°C. Tubes were then allowed to sit for 5 min on the bench, and the optical density of the center of the culture was measured.

### PCR and construct generation.

S. aureus strain USA300 LAC, cured of the 27-kb plasmid that confers antibiotic resistance, was used as the WT strain for mutant generation, unless otherwise stated. Primers used in this study are listed in Table 2. For mobilizing transposon insertion mutations into various genetic backgrounds, phage transduction was performed according to standard techniques. Phage lysate was prepared from the donor strain using phage 80α, recipient strains were infected, and transductants were selected using appropriate antibiotics (10). Insertions were confirmed by PCR. Markerless deletions were constructed using the pIMAY system, as previously described (32). For complementation, the full-length genes were amplified using primers listed in Table 2, ligated into pALC2073, and transformed into E. coli. All plasmids were passaged through RN4220 prior to transfer to the strain of interest.

**TABLE 2:** Primers used in this study^*a*^

Function	Primer name	Primer sequence^*a*^
Amplifying whole-length *purK* for complementation	purK F	GGGGAGCTCCAAAAAGTGGAGGACATGCAAAATG
	purK R	GGGGAATTCTGTCATGCTTTAATTACTCCCCTCA
Generating an upstream fragment for *purR* deletion	purR Up F	GGGGTCGACTTTTTGATATAGGGGCGAGTT
	purR Up R	CTTTTCAACCCTTCTATCCTA
Generating an downstream fragment for *purR* deletion	purR downF	TAGGATAGAAGGGTTGAAAAGAAGGAGTTTTAGTATTATGA
	purR downR	GGGGGTACCGTATATATCTCTCTGTTTTAT

a Underlined sequences indicate restriction sites.

### Mouse infections.

All animal experiments were performed in compliance with guidelines set out by the Canadian Council on Animal Care. All animal protocols (protocol 2017-028) were reviewed and approved by the University of Western Ontario Animal Use Subcommittee, a subcommittee of the University Council on Animal Care. Six- to 8-week-old female BALB/c mice (Charles River Laboratories) were injected via tail vein with 100 μl of bacterial culture containing 1 × 10^7^ CFU of bacteria. To prepare the bacteria, strains were grown to an OD_600_ 2 to 2.5 in TSB, washed twice with PBS, and resuspended to the desired numbers in PBS. Infections were allowed to proceed for up to 96 h before animals were euthanized or when they met guidelines for early euthanasia. Organs were harvested in PBS plus 0.1% (vol/vol) Triton X-100 and homogenized in a Bullet Blender Storm (Next Advance, Troy, NY), using 2 runs of 5 min at setting 10 and metal beads. Dilutions of organ homogenates were plated on tryptic soy agar (TSA) for CFU enumeration.

## Supplementary Material

Supplemental file 1

Supplemental file 2

Supplemental file 3

Supplemental file 4

Supplemental file 5

Supplemental file 6

Supplemental file 7
