# Supplementary material for: De Novo Purine Biosynthesis Is Required for Intracellular Growth of Staphylococcus aureus and for the Hypervirulence Phenotype of a purR Mutant
Source: Infect Immun. 2020 Apr 20;88(5):e00104-20. doi: 10.1128/IAI.00104-20 (PMC7171247; doi:10.1128/IAI.00104-20)
Supplement: Supplemental file 1 [file IAI.00104-20-s0001.pdf]

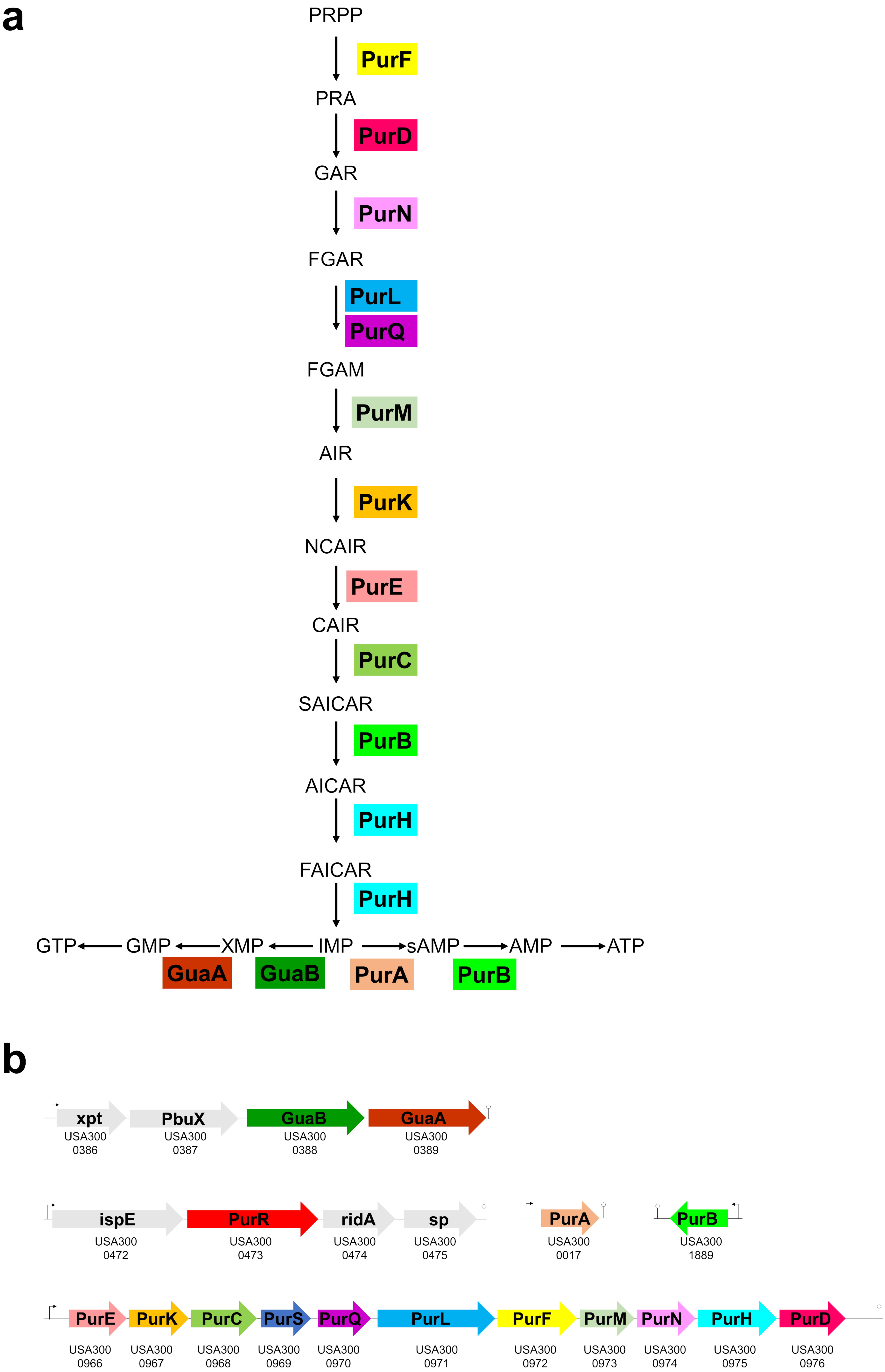

Supplementary Figure 1 – The purine biosynthesis pathway. A – schematic representation of the reactions that occur during de novo purine biosynthesis and the genes responsible. **B** – the genes responsible for de novo purine biosynthesis and control.
