# Supplementary material for: De Novo Purine Biosynthesis Is Required for Intracellular Growth of Staphylococcus aureus and for the Hypervirulence Phenotype of a purR Mutant
Source: Infect Immun. 2020 Apr 20;88(5):e00104-20. doi: 10.1128/IAI.00104-20 (PMC7171247; doi:10.1128/IAI.00104-20)
Supplement: Supplemental file 2 [file IAI.00104-20-s0002.pdf]

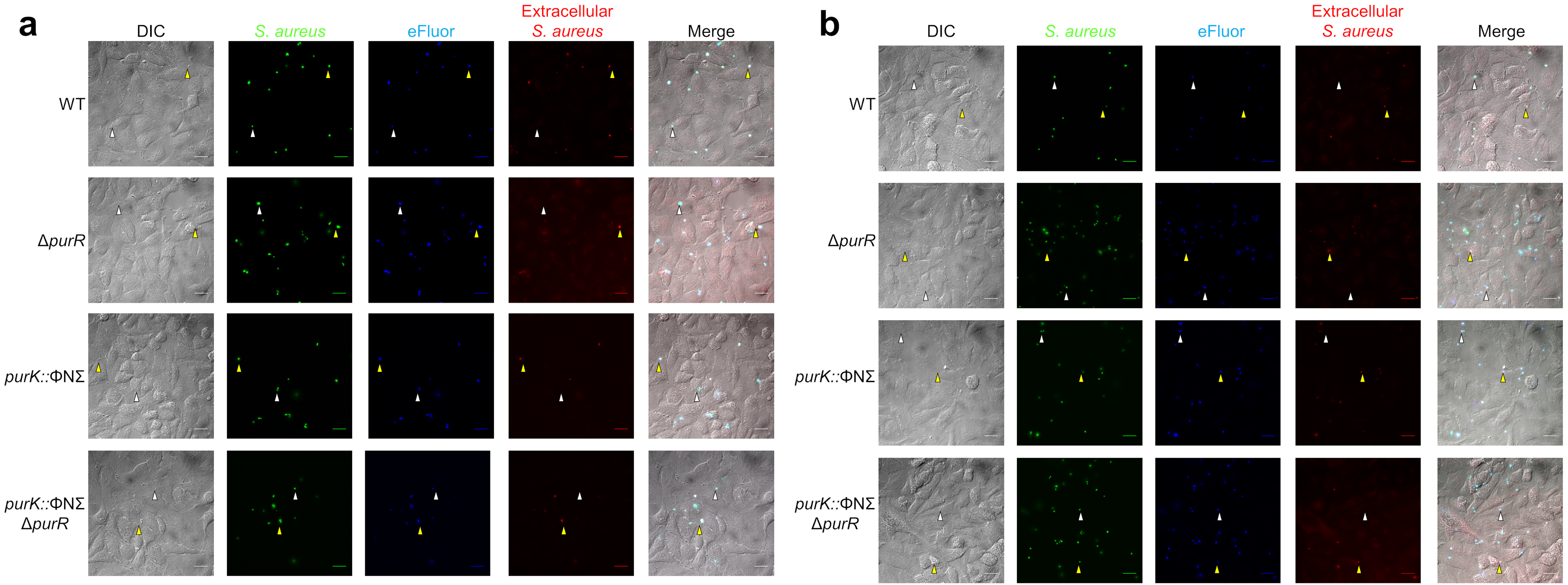

Supplementary Figure 2 - *pur* mutants have no defect in invasion of epithelial cells. Coverslips of cells were infected with bacteria grown to OD_{600} of 0.6 (**A**) or 2.0 (**B**) and stained after gentamicin treatment. At onset of infection, cells were stained with eFluor^{TM} 670 dye and prior to fixing were incubated with a Cy3 conjugated rabbit anti sheep IgG, to detect extracellular bacteria. Representative images are shown. White arrows indicate intracellular bacteria, yellow arrows indicated extracellular bacteria. Scale bar equals 20µm.
