# Supplementary material for: De Novo Purine Biosynthesis Is Required for Intracellular Growth of Staphylococcus aureus and for the Hypervirulence Phenotype of a purR Mutant
Source: Infect Immun. 2020 Apr 20;88(5):e00104-20. doi: 10.1128/IAI.00104-20 (PMC7171247; doi:10.1128/IAI.00104-20)
Supplement: Supplemental file 3 [file IAI.00104-20-s0003.pdf]

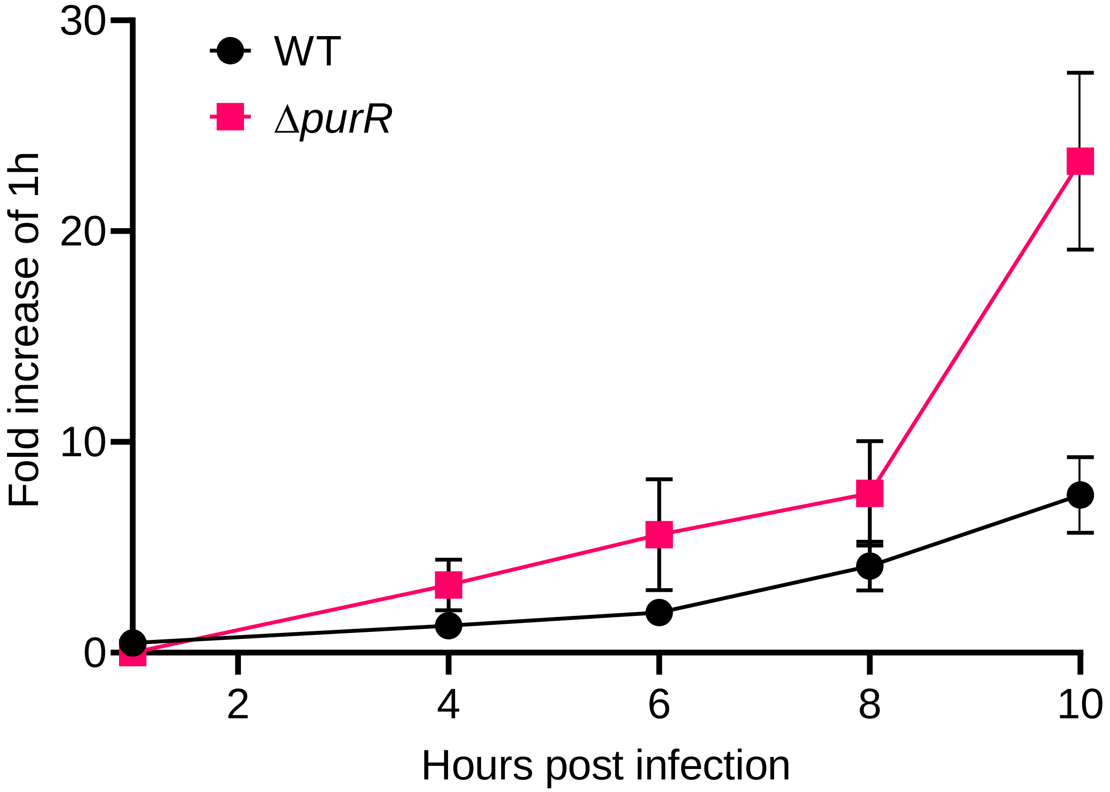

Supplementary Figure 3 – *S. aureus* intracellular replication cannot be detected before 8 hours post infection. A549 cells were infected with WT or ΔpurR S. aureus grown to OD_{600} of 0.6. At 4, 6, 8 and 10 hours post infection representative wells were lysed and CFU counted. Data shown are fold increase over samples recovered at 1 hpi (immediately after gentamicin treatment). Data shown are mean ± SEM of 4 experiments, with 2 biological replicates per experiment.
