# Supplementary material for: De Novo Purine Biosynthesis Is Required for Intracellular Growth of Staphylococcus aureus and for the Hypervirulence Phenotype of a purR Mutant
Source: Infect Immun. 2020 Apr 20;88(5):e00104-20. doi: 10.1128/IAI.00104-20 (PMC7171247; doi:10.1128/IAI.00104-20)
Supplement: Supplemental file 4 [file IAI.00104-20-s0004.pdf]

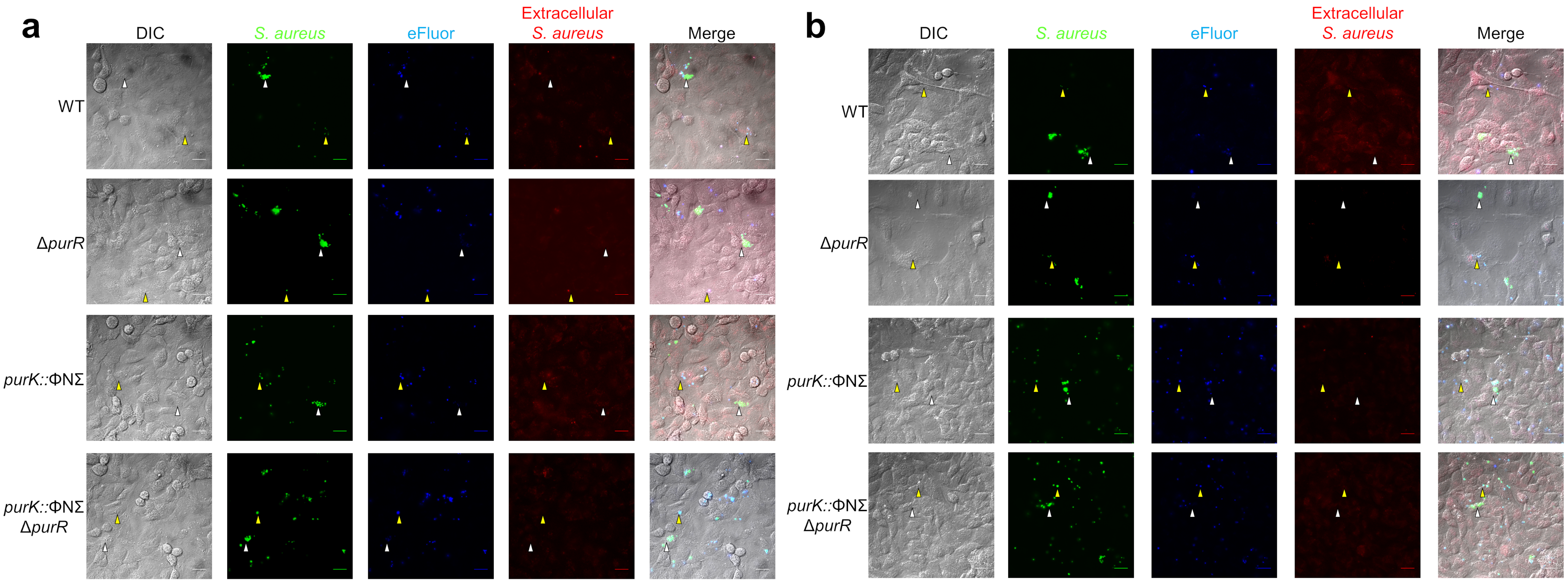

Supplementary Figure 4 - *pur* mutants show a defect in intracellular replication. Coverslips of cells were infected with bacteria grown to OD_{600} of 0.6 (**A**) or 2.0 (**B**) and stained at 10hpi. At onset of infection, cells were stained with eFluor^{TM} 670 dye and prior to fixing were incubated with a TRITC conjugated rabbit anti sheep IgG, to detect extracellular bacteria. Representative images are shown. White arrows indicat bacteria that have replicated, yellow arrows indicated bacteria that have not replicated. Scale bar equals 20µm.
