# Supplementary material for: De Novo Purine Biosynthesis Is Required for Intracellular Growth of Staphylococcus aureus and for the Hypervirulence Phenotype of a purR Mutant
Source: Infect Immun. 2020 Apr 20;88(5):e00104-20. doi: 10.1128/IAI.00104-20 (PMC7171247; doi:10.1128/IAI.00104-20)
Supplement: Supplemental file 5 [file IAI.00104-20-s0005.pdf]

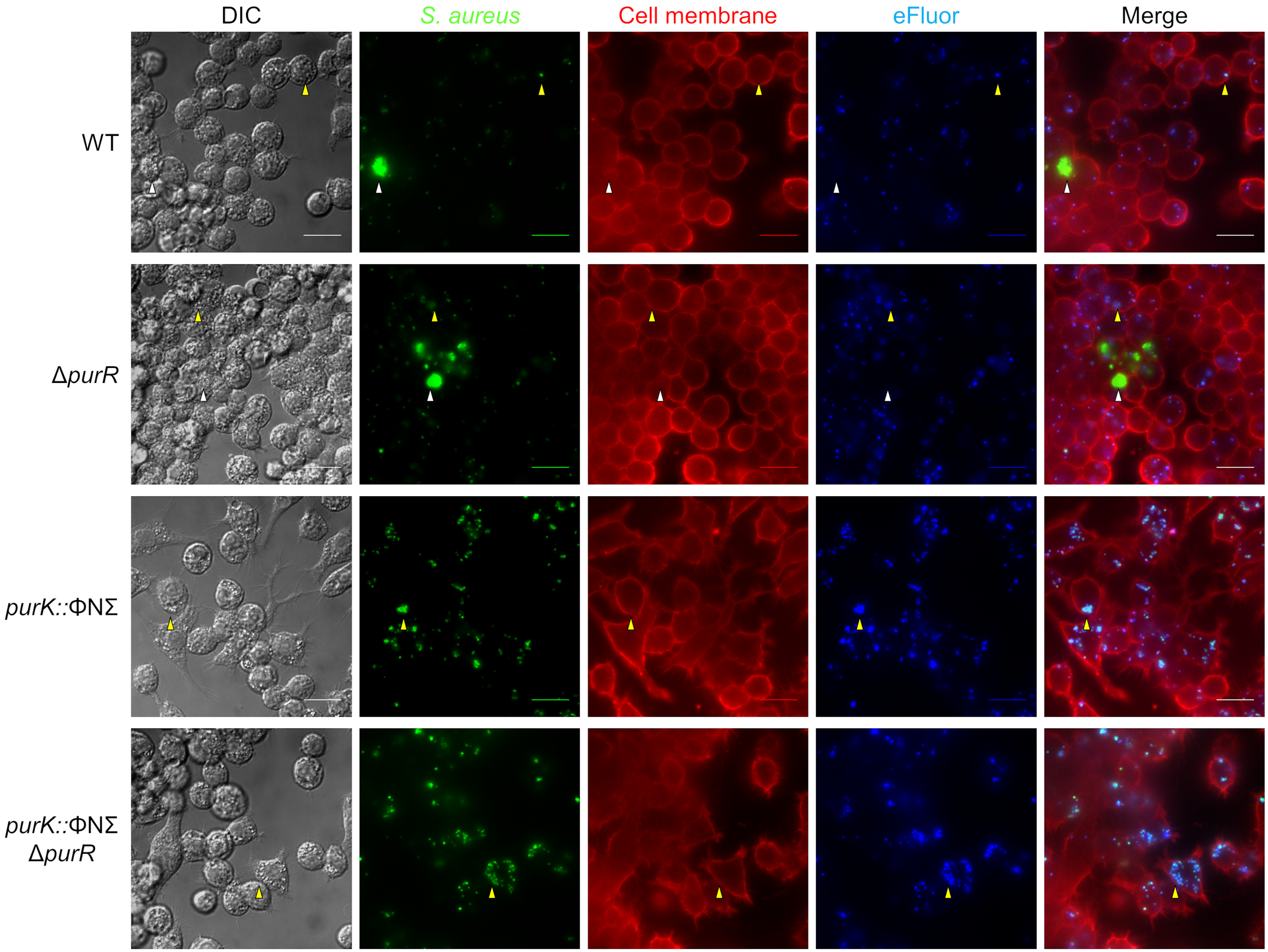

Supplementary Figure 5 - mutants in the *pur* operon cannot replicate in macrophages. Bacteria were labelled with eFluorTM 670 and used to infect cells, as in Figure 6A. At 18hpi the macrophage cell membrane was labelled with wheat germ agglutinin (WGA) for 5 min and the cells were fixed. Coverslips were imaged on a widefield microscope. Representative images are shown. Yellow arrows indicate bacteria that have not replicated, white arrows indicated bacteria that have replicated intracellularly (extracellular bacteria stain red). Scale bar equals 20μm.
