# Supplementary material for: De Novo Purine Biosynthesis Is Required for Intracellular Growth of Staphylococcus aureus and for the Hypervirulence Phenotype of a purR Mutant
Source: Infect Immun. 2020 Apr 20;88(5):e00104-20. doi: 10.1128/IAI.00104-20 (PMC7171247; doi:10.1128/IAI.00104-20)
Supplement: Supplemental file 6 [file IAI.00104-20-s0006.pdf]

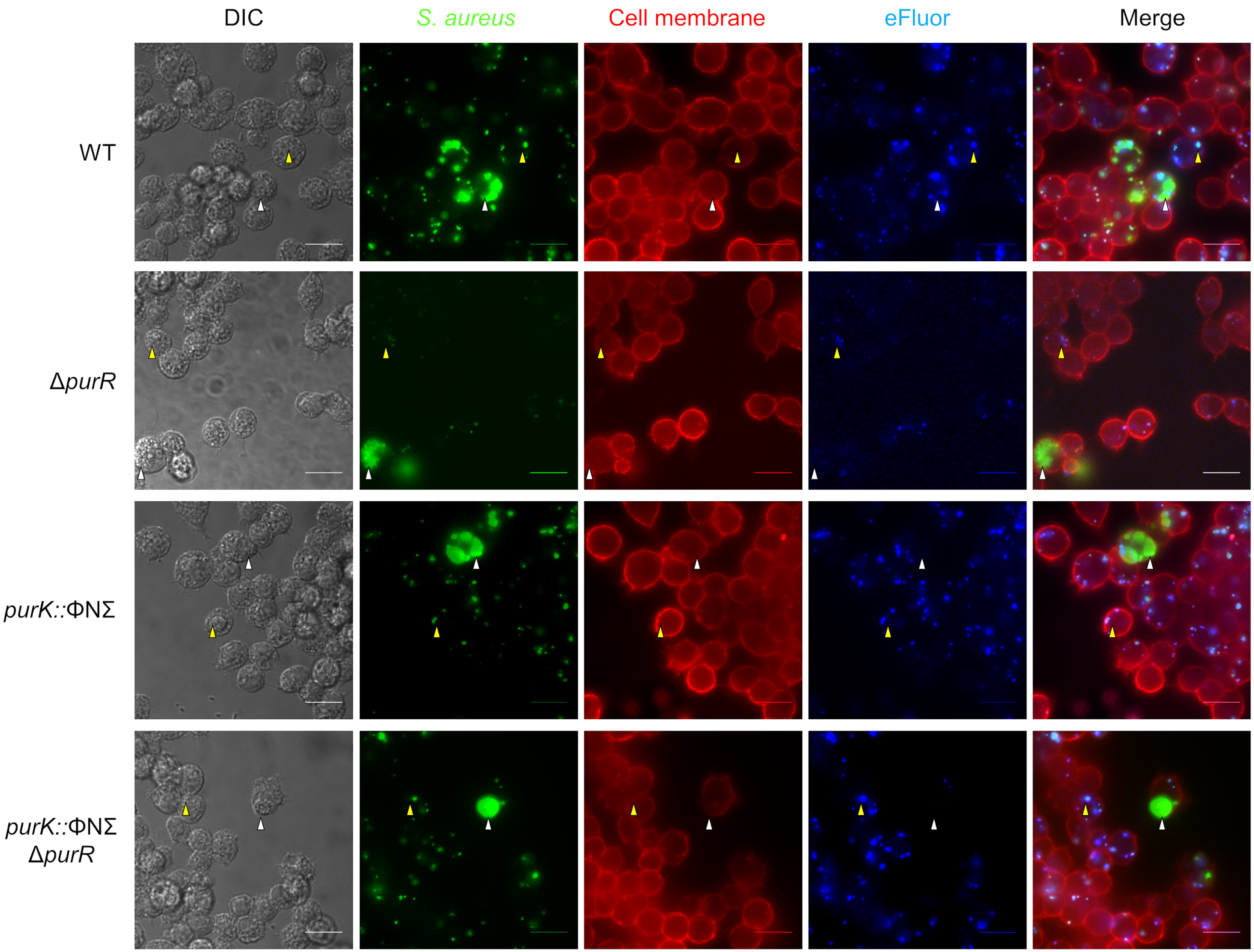

Supplementary Figure 6 - *pur* mutant intracellular growth is restored by the addition of exogenous purines. Bacteria were labelled with eFluor^{TM} 670 and used to infect cells, as in Figure 7A. At 18hpi the macrophage cell membrane was labelled with wheat germ agglutinin (WGA) for 5 min and the cells were fixed. Coverslips were imaged on a widefield microscope. Representative images are shown. Yellow arrows indicate bacteria that have not replicated, white arrows indicated bacteria that have replicated intracellularly (extracellular bacteria stain red). Scale bar equals 20µm.
