# Supplementary material for: De Novo Purine Biosynthesis Is Required for Intracellular Growth of Staphylococcus aureus and for the Hypervirulence Phenotype of a purR Mutant
Source: Infect Immun. 2020 Apr 20;88(5):e00104-20. doi: 10.1128/IAI.00104-20 (PMC7171247; doi:10.1128/IAI.00104-20)
Supplement: Supplemental file 7 [file IAI.00104-20-s0007.pdf]

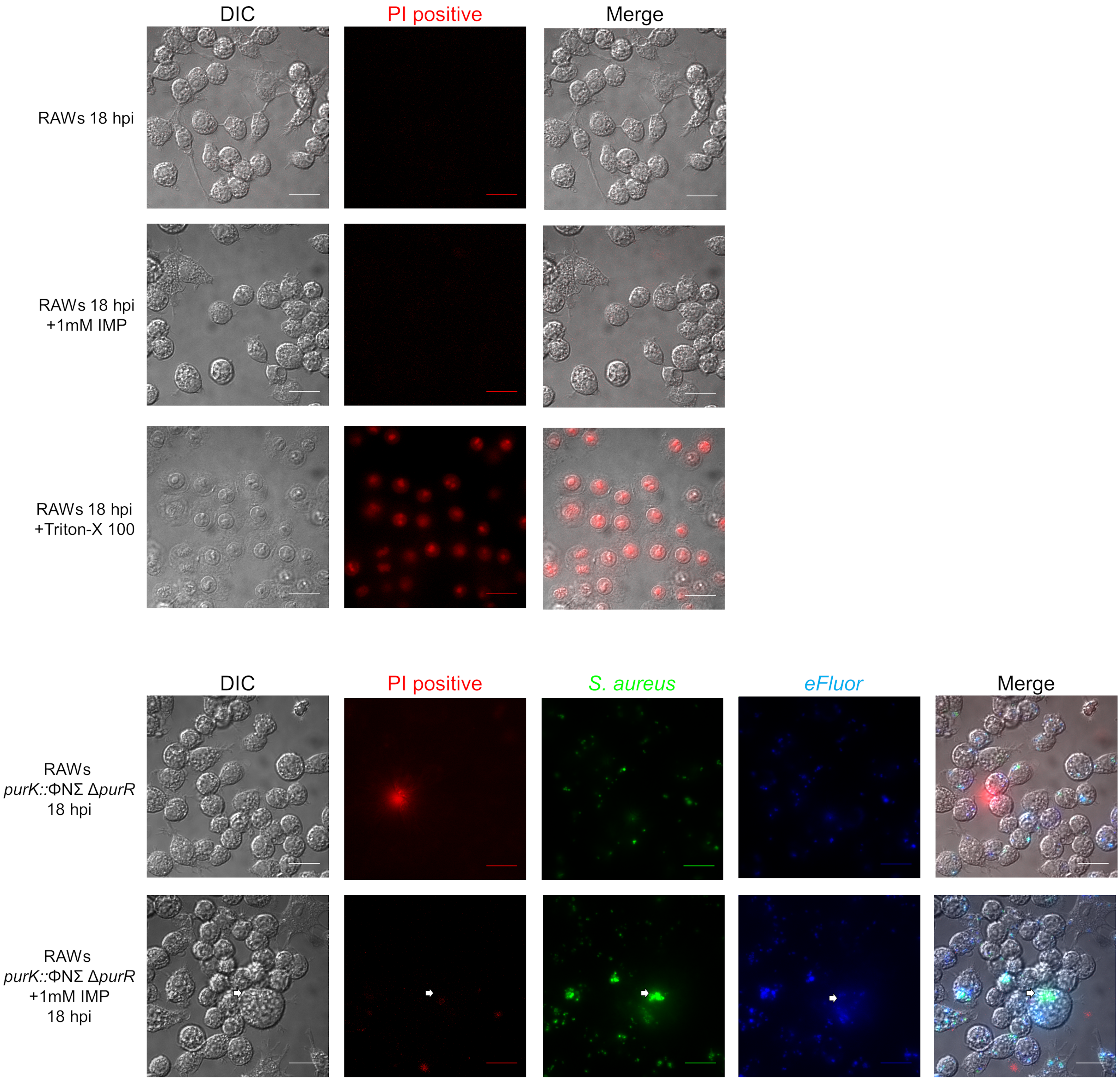

Supplementary Figure 7 - Exogenous purines restore the intracellular growth of a *pur* mutant without causing macrophages death. RAW 264.7 macrophages were mock infected or infected with a *purR purK* mutant of *S. aureus* for 18h. At 18hpi cells were treated with propidium iodide (PI) for 5 min to stain dead cells and imaged live on a widefield microscope. Bacteria were stained with eFluor^{TM} 670 at the onset of infection and bacteria that had undergone intracellular replication are indicated with the arrow. Representative images are shown. Scale bar equals 20µm.
